# Biomarkers of neuropsychiatric dysfunction in intensive care unit survivors: a prospective cohort study

**DOI:** 10.5935/2965-2774.20230422-en

**Published:** 2023

**Authors:** Franciani Rodrigues da Rocha, Renata Casagrande Gonçalves, Gabriele da Silveira Prestes, Danusa Damásio, Amanda Indalécio Goulart, Andriele Aparecida da Silva Vieira, Monique Michels, Maria Inês da Rosa, Cristiane Ritter, Felipe Dal-Pizzol

**Affiliations:** 1 Laboratory of Translational Biomedicine, Postgraduate Program in Health Sciences, Universidade do Extremo Sul Catarinense - Criciúma (SC), Brazil; 2 Laboratory of Experimental Pathophysiology, Postgraduate Program in Health Sciences, Health Sciences Unit, Universidade do Extremo Sul Catarinense - Criciúma (SC), Brazil; 3 Research Centre, Hospital São José - Criciúma (SC), Brazil

**Keywords:** Critical illness, Critical care outcomes, Cognitive dysfunction, Anxiety, Depression, Delirium, Patient discharge, Biomarkers, Intensive care units

## Abstract

**Objective:**

To assess factors associated with long-term neuropsychiatric outcomes, including biomarkers measured after discharge from the intensive care unit.

**Methods:**

A prospective cohort study was performed with 65 intensive care unit survivors. The cognitive evaluation was performed through the Mini-Mental State Examination, the symptoms of anxiety and depression were evaluated using the Hospital Anxiety and Depression Scale, and posttraumatic stress disorder was evaluated using the Impact of Event Scale-6. Plasma levels of amyloid-beta (1-42) [Aβ (1-42)], Aβ (1-40), interleukin (IL)-10, IL-6, IL-33, IL-4, IL-5, tumor necrosis factor alpha, C-reactive protein, and brain-derived neurotrophic factor were measured at intensive care unit discharge.

**Results:**

Of the variables associated with intensive care, only *delirium* was independently related to the occurrence of long-term cognitive impairment. In addition, higher levels of IL-10 and IL-6 were associated with cognitive dysfunction. Only IL-6 was independently associated with depression. Mechanical ventilation, IL-33 levels, and C-reactive protein levels were independently associated with anxiety. No variables were independently associated with posttraumatic stress disorder.

**Conclusion:**

Cognitive dysfunction, as well as symptoms of depression, anxiety, and posttraumatic stress disorder, are present in patients who survive a critical illness, and some of these outcomes are associated with the levels of inflammatory biomarkers measured at discharge from the intensive care unit.

## INTRODUCTION

With the advancement of assistance to critically ill patients, there was a decrease in mortality, leading to the need to analyze the impact of intensive care on long-term outcomes.^(1-5)^ Survivors of critical illness can develop postintensive care syndrome (PICS), a spectrum of conditions that include persistent cognitive dysfunction, acquired weakness, and psychiatric disorders,^(6)^ resulting in a decreased quality of life.^(7-9)^ Postintensive care syndrome can be defined as a new or worsening impairment in physical, cognitive or mental health status arising and persisting after hospitalization for critical illness,^(10)^ and global studies focus on one or two PICS parameters.^(6-9)^ These persistent physical, cognitive, and psychological deficiencies experienced by intensive care unit (ICU) survivors present relevant public health problems.^(11)^

There are reports that impairment of numerous neuropsychiatric domains that can directly and negatively affect the patient’s function is seen post-ICU discharge.^(11)^ The presence of depression, anxiety, posttraumatic stress disorder (PTSD), and cognitive impairments results in physical, psychological, and sometimes financial damage to patients, thereby deteriorating their quality of life.^(12-14)^

A recent meta-analysis identified 60 risk factors for the development of PICS, of which 33 were categorized as personal and 27 as ICU-related.^(15)^ Interestingly, most risk factors for neuropsychiatric impairments were not related to ICU care itself but to premorbid patient characteristics.^(11)^ Additionally, some of the proposed mechanisms for PICS overlap with other chronic diseases, such as cardiovascular disease, depression, and dementia, and from this perspective, inflammation, neurotrophic factors, and amyloid-beta (Aβ) would be major target candidates to be PICS biomarkers.^(16-18)^

Since the origin of PICS is multifactorial, the analysis of biomarkers can provide valuable information on the underlying mechanisms.^(18)^ Previous research has linked inflammation to the development of acute brain dysfunction.^(19-21)^ Focusing on PICS, some studies collected biomarkers at ICU admission and associated them with long-term outcomes.^(22-24)^ There are few reports associating biomarkers after acute disease resolution (i.e., after ICU or hospital discharge),^(25,26)^ and long-term outcomes. Inflammation at ICU discharge can independently be associated with one-year mortality in septic patients.^(26)^ Additionally, we demonstrated in a retrospective study that elevated circulating interleukin (IL)-6 and IL-10 concentrations at hospital discharge were associated with long-term cognitive dysfunction in ICU survivors.^(25)^

Therefore, this study aimed to assess factors associated with long-term neuropsychiatric outcomes, including biomarkers measured after discharge from the ICU. We hypothesize that even after discharge from the ICU, inflammation, neurotrophic factors, and Aβ still have an impact on long-term neuropsychiatric outcomes.

## METHODS

This was a single-center prospective cohort study approved by the Institutional Review Boards of our university (protocol 1.993.271) and hospital (protocol 1.824.369). All patients or their surrogates provided written consent before study inclusion.

### Setting and patients

The sample of the present study consisted of all patients who were admitted to a 20-bed ICU from a tertiary care, University-associated Hospital in southern Santa Catarina State, Brazil, from January 1, 2017, to December 31, 2017. The inclusion criteria were as follows: patients aged > 18 years who stayed in the ICU for ≥ 72 hours (medical or urgent surgery admissions) or ≥ 120 hours (elective surgery admissions), hospitalized within 24 - 120 hours after ICU discharge, and those who provided consent to participate in the study. Exclusion criteria were as follows: patients transferred from another ICU, ICU discharge to home or another hospital, admitted to the ICU due to exclusive palliative care or neurologic causes, and previous neurodegenerative disease.

### Procedures

All patients who were discharged from the ICU were screened daily, and those who met the inclusion criteria were considered eligible. The patient was invited to participate in the study from 24 to 120 hours after ICU discharge. At this time, sociodemographic characteristics, ICU admission, and ICU intervention data were collected. Disease severity was assessed by the Simplified Acute Physiology Score (SAPS) III score. Organ dysfunction was assessed by the Sequential Organ Failure Assessment (SOFA) score. Comorbidities were integrated into the Charlson comorbidity index, which included nineteen comorbidities in a weighted index that predicts the risk of death within 1 year of hospitalization. *Delirium* was measured using the Confusion Assessment Method for ICU (CAM-ICU) as part of the usual patient care. Furthermore, the referred diagnosis of anxiety and depression and the Barthel index prior to ICU admission were collected. Additionally, 5mL of blood was collected for the measurement of biomarkers.

Four months after hospital discharge, cognition and symptoms of anxiety and depression were assessed in the university outpatient clinic.

### Cognitive assessment

The Mini-Mental State Examination (MMSE) was performed to assess cognitive function. The following cutoff scores were used to classify patients as having cognitive deficiency: < 24 with higher education; < 23 with 6 to 12 years of study; < 22 with less than 6 years of study; and < 21 for illiterates.^(27)^

### Symptoms of anxiety and depression

This was assessed using the Hospital Anxiety and Depression Scale (HADS).^(28,29)^ For the anxiety subscale (HADS-A) and depression subscale (HADS-D), the following cutoff scores were considered: 0 - 7 points: unlikely anxiety or depression; 8 - 11 points: possible anxiety or depression; 12 - 21 points: likely anxiety or depression. Thus, depression and anxiety were defined as HADS ≥ 8 points.

### Posttraumatic stress disorder

Posttraumatic stress disorder was assessed using the Impact of Event Scale-6 (IES-6) with a cutoff score of 1.75.^(30)^

### Biomarker determination

Plasma levels of Aβ (1-42), Aβ (1-40), IL-10, IL-6, IL-33, IL-4, IL-5, tumor necrosis factor alpha (TNF-α), C-reactive protein (CRP), and brain-derived neurotrophic factor (BDNF) were evaluated using R&D ELISA kits. All markers, except CRP, were expressed as pg/mL. C-reactive protein was expressed as ng/mL.

### Statistical analysis

Inferential analysis of the data was performed using Statistical Package for the Social Sciences (SPSS), version 17.0. Continuous variables are summarized as the mean ± standard deviation (SD) or median and interquartile range (IQR). The homogeneity of variances was assessed by the Levene test. Categorical variables are presented as numbers and percentages and were compared using chi-square tests. Binary regression was used to assess the independent risk factors for outcomes. The model only included variables that had a p value of < 0.25 in the univariate analysis. Since mechanical ventilation, sedation use, and *delirium* had a clinically relevant association (Cramer´s V statistic 0.83, p < 0.00001 between mechanical ventilation and sedation, 0.50, p = 0.001 between mechanical ventilation and *delirium*, and 0.38, p = 0.012 between sedation and *delirium*), the variable with a lower p value for every single outcome in the univariate analysis was entered in the final model. The results from the univariate analysis are presented as p values, and those from binary regression are presented as relative risks and 95% confidence intervals. In all analyses, a p value of < 0.05 was considered to indicate statistical significance.

## RESULTS

**Figure 1:**
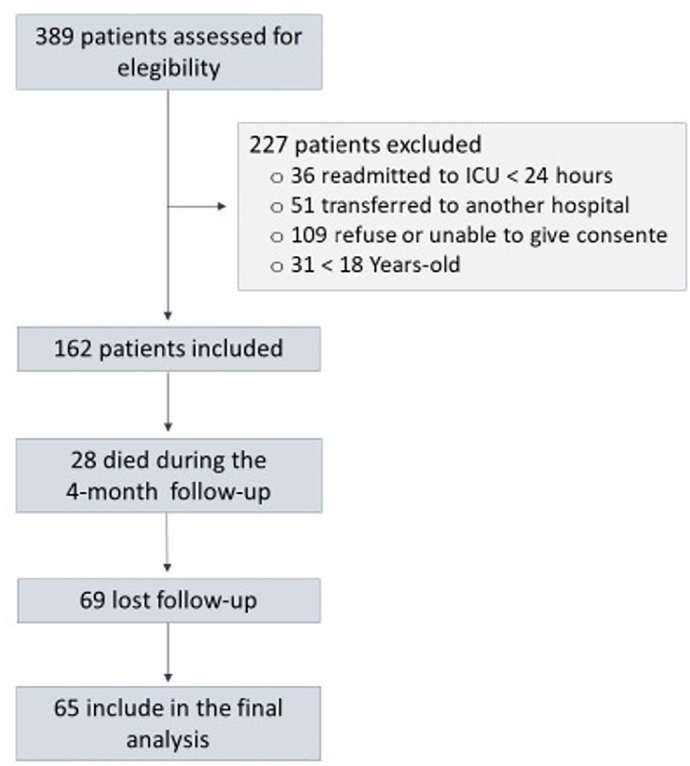
Flowchart of included patients.

From January 2017 to December 2017, a total of 389 patients were screened after ICU discharge (Figure 1). From these, 227 patients were excluded: 36 were readmitted to the ICU within 24 hours, 31 were aged < 18 years old, 51 were discharged to another hospital within 24 hours and 109 were unable or refused to give consent. Of the remaining 162 patients, 28 died during the 4-month follow-up, and another 69 patients were lost to follow-up. Thus, at the end of the 4-month follow-up, 65 patients were included in the analysis of cognitive dysfunction, depression, anxiety, and PTSD. General characteristics from the whole sample are presented in table 1. When comparing the baseline characteristics of the 69 patients who were lost to follow-up to those 65 included patients, no significant difference was found (data not shown).

**Table 1:** General characteristics of the included patients

Characteristics	
Age (years)	53 ± 17
Sex, male	40 (62)
Charlson score	1.94 ± 1.7
Admission type	
Clinical	38 (58)
Surgical, planned	1 (1.5)
Surgical, unplanned	26 (40)
SAPS III	59 ± 13
SOFA	3.5 ± 2.6
Vasopressor, yes	34 (52)
Sedation, yes	19 (29)
Mechanical ventilation, yes	28 (43)
*Delirium*, yes (%)	9 (14)
Nosocomial infection, yes	16 (25)
Length of ICU stay	5 (3 - 10)
Previous diagnosis of anxiety, yes	15 (23)
Previous diagnosis of depression, yes	19 (29)
Barthel index	91 ± 15

ICU - Intensive care unit; SAPS - Simplified Acute Physiologic Score; SOFA - Sequential Organ Failure Assessment. Results expressed as mean ± standard deviation, n (%) or median (interquartile range).

ICU - intensive care unit.

The first outcome evaluated was the presence of cognitive dysfunction, and 23 (35%) patients presented with cognitive dysfunction (Table 2). Among the variables associated with intensive care, only *delirium* was significantly related to the occurrence of long-term cognitive dysfunction (p = 0.034). In addition, elevated levels of IL-10 (p = 0.007) and IL-5 (p = 0.044) were associated with cognitive dysfunction in the univariate analysis. In the regression analysis, the only clinical variable independently associated with cognitive dysfunction was the presence of *delirium* during the ICU stay. Three different inflammatory markers were independently associated with long-term cognitive dysfunction: IL-6, IL-10, and IL-5.

**Table 2:** Independent predictors of cognitive dysfunction four months after intensive care unit discharge

	Cognitive dysfunction		p value^*^	RR (95%CI)†
	No n = 42	Yes n = 23		
Age (years)	56 ± 16	48 ± 17	0.09	0.98 (0.94 - 1.02)
Sex, male	26 (62)	14 (61)	0.93	
Charlson score	2.05 ± 1.79	1.74 ± 1.54	0.49	
SAPS III score	59 ± 12	58 ± 16	0.97	
SOFA score	3.8 ± 2.7	3.1 ± 2.2	0.32	
Vasopressor, yes	23 (55)	11 (48)	0.59	
Sedation, yes	10 (24)	9 (39)	0.19	
Mechanical ventilation, yes	15 (36)	13 (57)	0.10	
*Delirium*, yes	3 (7)	6 (26)	0.034	9.1 (1.5 - 54)
Nosocomial infection, yes	9 (21)	7 (30)	0.42	
Previous diagnosis of anxiety, yes	11 (26)	4 (17)	0.42	
Previous diagnosis of depression, yes	12 (28)	7 (30)	0.87	
Previous Barthel index	92 ± 14	90 ± 16	0.75	
Aβ 1-42	49 ± 61	46 ± 41	0.79	
Aβ 1-40	12 ± 17	16 ± 31	0.53	
IL-10	28 ± 8	43 ± 33	0.007	1.07 (1.02 - 1.13)
IL-6	1,653 ± 1,278	2,387 ± 1,933	0.07	1 (1 - 1.001)
IL-33	84 ± 3.5	85 ± 5.9	0.44	
TNF	8.5 ± 6.3	7 ± 1.7	0.28	
IL-4	70 ± 65	85 ± 53	0.35	
IL-5	10 ± 7.3	16 ± 13	0.044	1.07 (1 - 1.16)
CRP	47 ± 64	32 ± 28	0.31	
BDNF	109 ± 39	110 ± 39	0.87	

RR - relative risk; CI - confidence interval; SAPS - Simplified Acute Physiologic Score; SOFA - Sequential Organ Failure Assessment; Aβ - amyloid-beta; IL - interleukin; TNF - tumor necrosis factor; CRP - C-reactive protein; BDNF - brain-derived neurotrophic factor; % - percentage of the subgroup total.

* Based on a univariate analysis; † based on a binary regression model that included age, *delirium*, IL-10, IL-6 and IL-5. Results expressed as mean ± standard deviation and n (%).

Of the 65 patients, 40 (62%) had depression 4 months after discharge. No single care-related variable and only one biomarker (IL-6) were associated with depression in the univariate analysis (Table 3). Interestingly, only IL-6 levels were independently associated with depression in our sample. Furthermore, 37 (57%) patients presented with anxiety, and only mechanical ventilation and IL-33 and CRP levels were significantly associated with this outcome in the univariate analysis (Table 4). However, none of the variables were independently associated with anxiety in this sample.

**Table 3:** Independent predictors of depression four months after intensive care unit discharge

	Depression		p value^*^	RR (95%CI)†
	No n = 25	Yes n = 40		
Age (years)	54 ± 17	53 ± 17	0.76	
Sex, male	16 (64)	24 (60)	0.74	
Charlson score	1.88 ± 1.87	1.98 ± 1.6	0.82	
SAPS	58 ± 15	59 ± 12	0.75	
SOFA	3.7 ± 2.6	3.3 ± 2.5	0.59	
Vasopressor, yes	10 (40)	24 (60)	0.11	0.42 (0.13 - 1.36)
Sedation, yes	8 (32)	10 (25)	0.69	
Mechanical ventilation, yes	11 (44)	17 (42)	0.90	
*Delirium*, yes	2 (8)	7 (18)	0.28	
Nosocomial infection, yes	9 (36)	7 (17)	0.09	2.6 (0.75 - 9)
Previous diagnosis of anxiety, yes	4 (16)	11 (28)	0.28	
Previous diagnosis of depression, yes	7 (28)	12 (30)	0.86	
Previous Barthel index	90 ±17	93 ± 10	0.38	
Aβ 1-42	48 ± 39	48 ±63	0.96	
Aβ 1-40	12 ± 25	14 ± 21	0.70	
IL-10	35 ± 27	32 ± 18	0.62	
IL-6	1,583 ± 1,263	2,440 ± 1,868	0.031	1 (1 - 1.001)
IL-33	84 ± 5.1	84 ± 4.1	0.89	
TNF	7.2 ± 1.9	8.4 ± 6.4	0.36	
IL-4	88 ± 78	67 ± 47	0.24	1.00 (0.99 - 1.01)
IL-5	12 ± 8.4	12 ± 10	0.81	
CRP	29 ± 21	50 ± 66	0.07	0.99 (0.97 - 1.01)
BDNF	106 ± 28	111 ± 44	0.59	

RR - relative risk; CI - confidence interval; SAPS - Simplified Acute Physiologic Score; SOFA - Sequential Organ Failure Assessment; Aβ - amyloid-beta; IL - interleukin; TNF - tumor necrosis factor; CRP - C-reactive protein; BDNF - Brain-derived neurotrophic factor; % - percentage of the subgroup total.

* Based on a univariate analysis; † based on a binary regression model that included vasopressor, nosocomial infection, IL-6, IL-4 and C-reactive protein. Results expressed as mean ± standard deviation and n (%).

**Table 4:** Independent predictors of anxiety four months after intensive care unit discharge

	Anxiety		p value^*^	RR (95%CI)†
	No n = 28	Yes n = 37		
Age (years)	52 ± 18)	54 ± 16)	0.54	
Sex, male	16 (57)	24 (65)	0.52	
Charlson score	1.68 ± 1.54	2.14 ± 1.8	0.28	
SAPS	60 ± 14	58 ± 13	0.53	
SOFA	3.6 ± 2.5	3.4 (2.6	0.68	
Vasopressor, yes	14 (50)	20 (54)	0.74	
Sedation, yes	6 (21)	13 (35)	0.22	
Mechanical ventilation, yes	16 (57)	12 (32)	0.046	3.1 (0.95 - 10)
*Delirium*, yes	3 (10)	6 (16)	0.52	
Nosocomial infection, yes	5 (18)	11 (30)	0.27	
Previous diagnosis of anxiety, yes	6 (21)	9 (24)	0.78	
Previous diagnosis of depression, yes	7 (25)	12 (32)	0.51	
Previous Barthel index	92 ± 17	91 ± 12	0.64	
Aβ 1-42	44 ± 43	51 ± 63	0.60	
Aβ 1-40	13 ± 18	14 ± 26	0.95	
IL-10	34 ± 25	33 ± 19	0.89	
	**Anxiety**		p value^*^	**RR (95%CI)†**
	**No** **n = 28**	**Yes** **n = 37**		
IL-6	1,605 ± 1,233	2,145 ± 1,760	0.17	1.00 (0.99 - 1.00)
IL-33	83 ± 3.3	85 ± 5.1	0.043	0.86 (0.74 - 1.01)
TNF	6.9 ± 1.4	8.8 ± 6.7	0.096	0.87 (0.68 - 1.01)
IL-4	78 ± 69	74 ± 55	0.8	
IL-5	12 ± 8.9	12 ± 10	0.88	
CRP	28 ± 23	52 ± 68	0.049	0.98 (0.96 - 1.0)
BDNF	115 ± 53	104 ± 23	0.31	

RR - relative risk; CI - confidence interval; SAPS - Simplified Acute Physiologic Score; SOFA - Sequential Organ Failure Assessment; Aβ - amyloid-beta; IL - interleukin; TNF - tumor necrosis factor; CRP - C-reactive protein; BDNF - Brain-derived neurotrophic factor; % - percentage of the subgroup total.

* Based on a univariate analysis; † based on a binary regression model that included mechanical ventilation, IL-6, IL-33, tumor necrosis factor and C-reactive protein. Results expressed as mean ± standard deviation and n (%).

Thirteen (20%) survivors presented with symptoms of PTSD; however, no measured variable was associated with PTSD in either the univariate or multivariate analyses (Table 5).

**Table 5:** Independent predictors of posttraumatic stress disorder four months after intensive care unit discharge

	Posttraumatic stress disorder		p value^*^	RR (95%CI)†
	No n = 52	Yes n = 13		
Age (years)	55 ± 17	47 ± 15	0.15	0.98 (0.94 - 1.02)
Sex, male	32 (62)	8 (62)	1.0	
Charlson score	1.96 ± 1.83	1.85 ± 1.06	0.82	
SAPS score	59 ± 14	55 ± 9	0.18	0.98 (0.93 - 1.03)
SOFA	3.3 ± 2.1	4.3 ± 3.9	0.38	
Vasopressor, yes	28 (54)	6 (46)	0.61	
Sedation, yes	15 (29)	4 (31)	0.89	
Mechanical ventilation, yes	22 (42)	6 (46)	0.80	
*Delirium*, yes	8 (15)	1 (8)	0.47	
Nosocomial infection, yes	14 (27)	2 (15)	0.38	
Previous diagnosis of anxiety, yes	11 (21)	4 (30)	0.46	
Previous diagnosis of depression, yes	16 (31)	3 (23)	0.58	
Previous Barthel index	93 ± 14	85 ± 18	0.20	0.97 (0.94 - 1.01)
Aβ 1-42	46 ± 58	55 ± 42	0.63	
Aβ 1-40	14 ± 25	12 ± 12	0.83	
IL-10	32 ± 17	40 ± 35	0.24	1.01 (0.98 - 1.03)
IL-6	1,512 ± 817	2,013 ± 1,695	0.30	
IL-33	84 ± 4.6	84 ± 4.0	0.57	
TNF	7.9 ± 5.7	8.1 ± 1.7	0.95	
IL-4	75 ± 64	76 ± 49	0.99	
IL-5	13 ± 10	11 ± 7.7	0.50	
CRP	46 ± 59	26 ± 24	0.25	
BDNF	110 ± 42	106 ± 16	0.75	

RR - relative risk; CI - confidence interval; SAPS - Simplified Acute Physiologic Score; SOFA - Sequential Organ Failure Assessment; Aβ - amyloid-beta; IL - interleukin; TNF - tumor necrosis factor; CRP - C-reactive protein; BDNF - brain-derived neurotrophic factor; % - percentage of the subgroup total.

* Based on a univariate analysis; † based on a binary regression model that included age, SAPS, and previous Barthel Index. Results expressed as mean ± standard deviation and n (%).

## DISCUSSION

Here, we demonstrated that in addition to variables related to critical illness, inflammatory biomarkers were also related to these long-term outcomes, even when collected after ICU discharge. This is different from previous studies wherein blood was collected in the initial days of ICU admission and could provide new insights into how persistent low-grade inflammation observed in survivors would impact long-term outcomes,^(9)^ which would help to better understand and design trials aimed at preventing or treating PICS.^(31,32)^

The mechanisms involved in late neurocognitive changes include inflammation and neuronal apoptosis, which consequently cause cerebral atrophy.^(33,34)^ It is believed that systemic insults of critical illnesses can lead to damage to the blood‒brain barrier, consequently resulting in neuroinflammation and acute neuronal injury,^(35)^ Evidence points to the association of plasma biomarkers of inflammation, endothelial dysfunction, damage to the blood‒brain barrier, and neuronal damage with the presence of *delirium*,^(36-38)^ and *delirium* was recently associated with long-term outcomes.^(39,40)^ Here, we demonstrated that three different inflammatory markers were independently associated with long-term cognitive impairment (IL-6, IL-10, and IL-5). IL-6 and IL-10 are frequently related to long-term outcomes in critically ill patients, including mortality,^(41)^ cardiovascular disease,^(42)^ and cognitive impairment.^(25)^ It was unexpected that IL-5 levels were associated with cognitive dysfunction. IL-5 is a prototypical T helper cell type 2 (Th2) cytokine, and IL-33 is believed to have a protective effect on brain inflammation and cognitive decline.^(43)^ Another intriguing factor is that Aβ (1-40) and Aβ (1-42) are not related to long-term cognitive impairment. It was expected from animal models that an increase in these markers would be related to long-term cognitive function.^(44,45)^ Inflammation and formation of Aβ is a well-known phenomenon; however, we could not observe this in our cohort, and Aβ (1-40) and Aβ (1-41) levels were associated with the risk of dementia.^(46)^

Psychological morbidity is persistent, and the observed symptoms of depression and anxiety in these patients can negatively impact their quality of life. IL-6 was independently associated with depression; however, anxiety was associated with IL-33 and CRP levels. Furthermore, no biomarker was associated with PTSD. At least in animal models, anxiety and depression are strongly associated events.^(47)^ Chronic mild stress causes both anxiety and depression, is associated with long-term cognitive dysfunction, and potentiates the dysfunction observed in septic survivors.^(48,49)^ It is believed that chronic stress and inflammation combine to compromise vascular and brain function. The resulting increases in proinflammatory cytokines and microglial activation drive brain pathology, leading to depression and mild cognitive impairment.^(50)^ Unfortunately, we could not determine a clear relationship between cytokines and both depressive and anxious states 4 months after hospital discharge. This either indicates that these are nonrelated dysfunctions in this population, or it only indicates a limitation of our study and should be further evaluated.

Some aspects of our study should be noted. First, approximately 50% loss to follow-up was observed during the 4-month follow-up period, and we likely missed more disabled patients who could not visit our outpatient clinic. However, baseline characteristics were similar when comparing these two groups of patients. Second, it was decided that blood should be collected after ICU discharge; thus, the measured biomarkers do not reflect the acute inflammatory response related to critical illness but probably are an indicator of the chronic low-grade inflammation observed in survivors, thus resulting in different pathophysiological implications when compared with the results of other studies. Ideally, blood collection at ICU admission, ICU discharge, hospital discharge, and outpatient clinic evaluation would provide a more comprehensive understanding of the impact of biomarkers on long-term neuropsychological outcomes, and to this end, a multicenter effort is highly relevant. Third, cerebrospinal fluid (CSF) biomarkers may better reflect brain-specific modifications that could drive neuropsychiatric outcomes. However, the obtention of CSF is not routinely employed in the care of critically ill patients. In this context, the use of plasma biomarkers is more clinically relevant, despite the fact that it can lose some information only given by CSF biomarkers. Fourth, baseline assessment of the patient´s cognitive status and anxiety, depression or PTSD symptoms was not possible due to the nature of ICU conditions. Thus, we analyzed prevalent and not incident symptoms. This is a limitation intrinsic to almost every study in this field. Fifth, given the small number of events due to the limited sample size, the regression analysis may be underpowered; therefore, it is important to keep this limitation in mind when interpreting the results presented here.

## CONCLUSION

Cognitive dysfunction, as well as symptoms of depression, anxiety, and posttraumatic stress disorder, are present in patients who survive a critical illness. However, although inflammation was a common pathway between all outcomes measured, there was no single common biomarker that predicted brain dysfunctions measured in this study.

## References

[1] Davydow DS, Gifford JM, Desai SV, Bienvenu OJ, Needham DM. (2009). Depression in general intensive care unit survivors: a systematic
review. Intensive Care Med.

[2] Desai SV, Law TJ, Needham DM. (2011). Long-term complications of critical care. Crit Care Med.

[3] Rawal G, Yadav S, Kumar R. (2017). Post-intensive care syndrome: an overview. J Transl Int Med.

[4] Paul N, Ribet Buse E, Knauthe AC, Nothacker M, Weiss B, Spies CD. (2023). Effect of ICU care bundles on long-term patient-relevant
outcomes: a scoping review. BMJ Open.

[5] Loss SH, Nunes DS, Franzosi OS, Salazar GS, Teixeira C, Vieira SR. (2017). Chronic critical illness: are we saving patients or creating
victims?. Rev Bras Ter Intensiva.

[6] Pereira S, Cavaco S, Fernandes J, Moreira I, Almeida E, Seabra-Pereira F (2018). Long-term psychological outcome after discharge from intensive
care. Rev Bras Ter Intensiva.

[7] Wintermann GB, Petrowski K, Weidner K, Strauß B, Rosendahl J. (2019). Impact of post-traumatic stress symptoms on the health-related
quality of life in a cohort study with chronically critically ill patients
and their partners: age matters. Crit Care.

[8] Ehlenbach WJ, Hough CL, Crane PK, Haneuse SJ, Carson SS, Curtis JR (2010). Association between acute care and critical illness
hospitalization and cognitive function in older adults. JAMA.

[9] Barichello T, Sayana P, Giridharan VV, Arumanayagam AS, Narendran B, Della Giustina A (2019). Long-term cognitive outcomes after sepsis: a translational
systematic review. Mol Neurobiol.

[10] Needham DM, Davidson J, Cohen H, Hopkins RO, Weinert C, Wunsch H (2012). Improving long-term outcomes after discharge from intensive care
unit: report from a stakeholders’ conference. Crit Care Med.

[11] Iwashyna TJ, Ely EW, Smith DM, Langa KM. (2010). Long-term cognitive impairment and functional disability among
survivors of severe sepsis. JAMA.

[12] Dinglas VD, Aronson Friedman L, Colantuoni E, Mendez-Tellez PA, Shanholtz CB, Ciesla ND (2017). Muscle weakness and 5-year survival in acute respiratory distress
syndrome survivors. Crit Care Med.

[13] Brück E, Schandl A, Bottai M, Sackey P. (2018). The impact of sepsis, delirium, and psychological distress on
self-rated cognitive function in ICU survivors-a prospective cohort
study. J Intensive Care.

[14] Brown SM, Bose S, Banner-Goodspeed V, Dinglas VD, Hopkins RO, Jackson JC, Mir-Kasimov M, Needham DM, Sevin CM, Addressing Post Intensive Care Syndrome 01 (APICS-01) study
team (2019). Approaches to addressing post-intensive care syndrome among
intensive care unit (ICU) survivors: a narrative review. Ann Am Thorac Soc.

[15] Lee M, Kang J, Jeong YJ. (2020). Risk factors for post-intensive care syndrome: a systematic
review and meta-analysis. Aust Crit Care.

[16] Fong TG, Davis D, Growdon ME, Albuquerque A, Inouye SK. (2015). The interface between delirium and dementia in elderly
adults. Lancet Neurol.

[17] Michels M, Michelon C, Damásio D, Vitali AM, Ritter C, Dal-Pizzol F. (2019). biomarker predictors of delirium in acutely ill patients: a
systematic review. J Geriatr Psychiatry Neurol.

[18] van den Boogaard M, Kox M, Quinn KL, van Achterberg T, van der Hoeven JG, Schoonhoven L (2011). Biomarkers associated with delirium in critically ill patients
and their relation with long-term subjective cognitive dysfunction;
indications for different pathways governing delirium in inflamed and
noninflamed patients. Crit Care.

[19] Maclullich AM, Ferguson KJ, Miller T, de Rooij SE, Cunningham C. (2008). Unravelling the pathophysiology of delirium: a focus on the role
of aberrant stress responses. J Psychosom Res.

[20] Ritter C, Tomasi CD, Dal-Pizzol F, Pinto BB, Dyson A, de Miranda AS (2014). Inflammation biomarkers and delirium in critically ill
patients. Crit Care.

[21] van Munster BC, Korevaar JC, Zwinderman AH, Levi M, Wiersinga WJ, De Rooij SE. (2008). Time-course of cytokines during delirium in elderly patients with
hip fractures. J Am Geriatr Soc.

[22] Hughes CG, Patel MB, Brummel NE, Thompson JL, McNeil JB, Pandharipande PP (2018). Relationships between markers of neurologic and endothelial
injury during critical illness and long-term cognitive impairment and
disability. Intensive Care Med.

[23] Darden DB, Brakenridge SC, Efron PA, Ghita GL, Fenner BP, Kelly LS (2021). Biomarker evidence of the persistent inflammation,
immunosuppression and catabolism syndrome (PICS) in chronic critical illness
(CCI) after surgical sepsis. Ann Surg.

[24] Mankowski RT, Anton SD, Ghita GL, Brumback B, Darden DB, Bihorac A (2022). Older adults demonstrate biomarker evidence of the persistent
inflammation, immunosuppression, and catabolism syndrome (PICS) after
sepsis. J Gerontol A Biol Sci Med Sci.

[25] Maciel M, Benedet SR, Lunardelli EB, Delziovo H, Domingues RL, Vuolo F (2019). Predicting long-term cognitive dysfunction in survivors of
critical illness with plasma inflammatory markers: a retrospective cohort
study. Mol Neurobiol.

[26] Soussi S, Sharma D, Jüni P, Lebovic G, Brochard L, Marshall JC, Lawler PR, Herridge M, Ferguson N, Del Sorbo L, Feliot E, Mebazaa A, Acton E, Kennedy JN, Xu W, Gayat E, Dos Santos CC, FROG-ICU; CCCTBG trans-trial group study for InFACT - the
International Forum for Acute Care Trialists (2022). Identifying clinical subtypes in sepsis-survivors with different
one-year outcomes: a secondary latent class analysis of the FROG-ICU
cohort. Crit Care.

[27] Rosa RG, Kochhann R, Berto P, Biason L, Maccari JG, De Leon P (2018). More than the tip of the iceberg: association between
disabilities and inability to attend a clinic-based post-ICU follow-up and
how it may impact on health inequalities. Intensive Care Med.

[28] Botega NJ, Bio MR, Zomignani MA, Garcia C Jr (1995). Pereira WA. [Mood disorders among inpatients in ambulatory and
validation of the anxiety and depression measurement scale
HAD]. Rev Saúde Publica.

[29] Marcolino JA, Mathias LA, Piccinini Filho L, Guaratini AA, Suzuki FM, Alli LA. (2007). Hospital Anxiety and Depression Scale: a study on the validation
of the criteria and reliability on preoperative patients. Rev Bras Anestesiol.

[30] Hosey MM, Leoutsakos JS, Li X, Dinglas VD, Bienvenu OJ, Parker AM (2019). Screening for posttraumatic stress disorder in ARDS survivors:
validation of the Impact of Event Scale-6 (IES-6). Crit Care.

[31] Dong CH, Gao CN, An XH, Li N, Yang L, Li DC (2021). Nocturnal dexmedetomidine alleviates post-intensive care syndrome
following cardiac surgery: a prospective randomized controlled clinical
trial. BMC Med.

[32] Wang S, Hammes J, Khan S, Gao S, Harrawood A, Martinez S (2018). Improving Recovery and Outcomes Every Day after the ICU
(IMPROVE): study protocol for a randomized controlled trial. Trials.

[33] Cunningham C. (2011). Systemic inflammation and delirium: important co-factors in the
progression of dementia. Biochem Soc Trans.

[34] Pandharipande PP, Girard TD, Jackson JC, Morandi A, Thompson JL, Pun BT, Brummel NE, Hughes CG, Vasilevskis EE, Shintani AK, Moons KG, Geevarghese SK, Canonico A, Hopkins RO, Bernard GR, Dittus RS, Ely EW, BRAIN-ICU Study Investigators (2013). Long-term cognitive impairment after critical
illness. N Engl J Med.

[35] Mazeraud A, Righy C, Bouchereau E, Benghanem S, Bozza FA, Sharshar T. (2020). Septic associated encephalopathy: a comprehensive
review. Neurotherapeutics.

[36] Hayhurst CJ, Patel MB, McNeil JB, Girard TD, Brummel NE, Thompson JL (2020). Association of neuronal repair biomarkers with delirium among
survivors of critical illness. J Crit Care.

[37] Liu X, Yu Y, Zhu S. (2018). Inflammatory markers in postoperative delirium (POD) and
cognitive dysfunction (POCD): a meta-analysis of observational
studies. PLoS One.

[38] Tomasi CD, Vuolo F, Generoso J, Soares M, Barichello T, Quevedo J (2017). Biomarkers of delirium in a low-risk community-acquired
pneumonia-induced sepsis. Mol Neurobiol.

[39] Hayhurst CJ, Marra A, Han JH, Patel MB, Brummel NE, Thompson JL (2020). Association of hypoactive and hyperactive delirium with cognitive
function after critical illness. Crit Care Med.

[40] Girard TD, Thompson JL, Pandharipande PP, Brummel NE, Jackson JC, Patel MB (2018). Clinical phenotypes of delirium during critical illness and
severity of subsequent long-term cognitive impairment: a prospective cohort
study. Lancet Respir Med.

[41] Yende S, D’Angelo G, Kellum JA, Weissfeld L, Fine J, Welch RD (2008). Inflammatory markers at hospital discharge predict subsequent
mortality after pneumonia and sepsis. Am J Respir Crit Care Med.

[42] Menéndez R, Méndez R, Aldás I, Reyes S, Gonzalez-Jimenez P, España PP (2019). Community-acquired pneumonia patients at risk for early and
long-term cardiovascular events are identified by cardiac
biomarkers. Chest.

[43] Fung IT, Sankar P, Zhang Y, Robison LS, Zhao X, D’Souza SS (2020). Activation of group 2 innate lymphoid cells alleviates
aging-associated cognitive decline. J Exp Med.

[44] Gasparotto J, Girardi CS, Somensi N, Ribeiro CT, Moreira JCF, Michels M (2018). Receptor for advanced glycation end products mediates
sepsis-triggered amyloid-β accumulation, Tau phosphorylation, and
cognitive impairment. J Biol Chem.

[45] Michelon C, Michels M, Abatti M, Vieira A, Borges H, Dominguini D (2020). The role of secretase pathway in long-term brain inflammation and
cognitive impairment in an animal model of severe sepsis. Mol Neurobiol.

[46] van Oijen M, Hofman A, Soares HD, Koudstaal PJ, Breteler MM. (2006). Plasma Abeta(1-40) and Abeta(1-42) and the risk of dementia: a
prospective case-cohort study. Lancet Neurol.

[47] Dal-Pizzol F, de Medeiros GF, Michels M, Mazeraud A, Bozza FA, Ritter C (2021). What animal models can tell us about long-term psychiatric
symptoms in sepsis survivors: a systematic review. Neurotherapeutics.

[48] Steckert AV, Dominguini D, Michels M, Abelaira HM, Tomaz DB, Sonai B (2017). The impact of chronic mild stress on long-term depressive
behavior in rats which have survived sepsis. J Psychiatr Res.

[49] Savi FF, de Oliveira A, de Medeiros GF, Bozza FA, Michels M, Sharshar T (2021). What animal models can tell us about long-term cognitive
dysfunction following sepsis: A systematic review. Neurosci Biobehav Rev.

[50] Hayley S, Hakim AM, Albert PR. (2021). Depression, dementia and immune dysregulation. Brain.

